# Supplementary material for: Determining the influential factors of postnatal care in Bangladesh using multilevel logistic regression
Source: PLoS One. 2024 Nov 7;19(11):e0313424. doi: 10.1371/journal.pone.0313424 (PMC11542818; doi:10.1371/journal.pone.0313424)
Supplement: Attachment — Submitted filename: Point-by-point response to reviewers.docx [file pone.0313424.s001.docx]

**Authors responses to the review comments:**

We would like to sincerely thank the anonymous reviewers, the Academic Editor, for their valuable comments. We have considered all comments and then thoroughly revised and formatted the manuscript. A detailed response to each comment is provided in the tables as follows.

| **Editor comments:** | **Response** | **Note** |
| --- | --- | --- |
| Please include the following items when submitting your revised manuscript:  A rebuttal letter that responds to each point raised by the academic editor and reviewer(s). You should upload this letter as a separate file labeled 'Response to Reviewers'.  A marked-up copy of your manuscript that highlights changes made to the original version. You should upload this as a separate file labeled 'Revised Manuscript with Track Changes'.  An unmarked version of your revised paper without tracked changes. You should upload this as a separate file labeled 'Manuscript'.  If you would like to make changes to your financial disclosure, please include your updated statement in your cover letter. Guidelines for resubmitting your figure files are available below the reviewer comments at the end of this letter. | Thanks. We appreciate your feedback. As per comments, a careful revision has been conducted, and all required files are uploaded to the journal submission system. | The revised texts are highlighted in “red” color. |
| **Journal Requirements:** | **Response** | **Note** |
| When submitting your revision, we need you to address these additional requirements.  1. Please ensure that your manuscript meets PLOS ONE's style requirements, including those for file naming. The PLOS ONE style templates can be found at  https://journals.plos.org/plosone/s/file?id=wjVg/PLOSOne_formatting_sample_main_body.pdf and  https://journals.plos.org/plosone/s/file?id=ba62/PLOSOne_formatting_sample_title_authors_affiliations.pdf | Thanks. We revised the format of the manuscript following the PLOS ONE style. | The revised texts are in “red”. Page: 1-16 |
| 2. Please provide additional details regarding participant consent. In the ethics statement in the Methods and online submission information, please ensure that you have specified (1) whether consent was informed and (2) what type you obtained (for instance, written or verbal, and if verbal, how it was documented and witnessed). If your study included minors, state whether you obtained consent from parents or guardians. If the need for consent was waived by the ethics committee, please include this information.  If you are reporting a retrospective study of medical records or archived samples, please ensure that you have discussed whether all data were fully anonymized before you accessed them and/or whether the IRB or ethics committee waived the requirement for informed consent. If patients provided informed written consent to have data from their medical records used in research, please include this information  Once you have amended this/these statement(s) in the Methods section of the manuscript, please add the same text to the “Ethics Statement” field of the submission form (via “Edit Submission”).  For additional information about PLOS ONE ethical requirements for human subjects research, please refer to http://journals.plos.org/plosone/s/submission-guidelines#loc-human-subjects-research. | Thanks. We add the Ethics Statement in the manuscript and submission system. | The revised texts are in “red”. Page: 7 |
| 3.Please include captions for your Supporting Information files at the end of your manuscript, and update any in-text citations to match accordingly. Please see our Supporting Information guidelines for more information: http://journals.plos.org/plosone/s/supporting-information. | Thanks. We have no supplementary files for this manuscript. |  |
| **Reviewer 1 comments:** | **Response** | **Note** |
| General comments:  The study is lacking novelty with absence of rationality, knowledge gap, and unsupported conclusions with findings. The authors should revise the introduction section to clearly outline the novelty and significance of their study. This should include a comprehensive rationale and a well-defined knowledge gap that their research aims to fill. The authors should carefully reassess their findings, ensuring that their conclusions are fully supported by the presented data. It is important to address any discrepancies and provide a more rigorous analysis and interpretation of the results. Additionally, I suggest that the authors strengthen the discussion section by highlighting the previous studies in different settings, and implications of the findings in order to enhance the overall quality of the manuscript. | Thank you very much for carefully checking the manuscript and providing comments and feedback. We believe that this helps to improve the quality of the manuscript. |  |
| Specific comments:  Abstract  - It lacks rationale for determining the contributing factors of postnatal care. Authors should add this.  - Objective does not support the conclusions.  - What is your suggestion based on study findings? | Thanks. We revised the Abstract section as per your comments. | The revised texts are in “red”. Page: 1-2 |
| Introduction:  - This section is severely disorganized.  - There is absence of significance of the study  - \Knowledge gap of the study is ;[not clear  - The authors stated that "However, most of the previous studies used logistic regression for finding the contributing factors of PNC. Because of the correlation, it is obligatory to consider cluster effect on regression models when performing postpartum care analysis that will yield efficient results than the general logistic regression model." What is the basis for claiming "Because of the correlation, it is obligatory to consider cluster effect on regression models when performing postpartum care analysis that will yield efficient results than the general logistic regression model"?. It is there any theoretical background or empirical evidence?  - This section is lacking the literature related to determinants of PNC in Bangladesh and other developing countries and the reflection of receiving PNC helps to lessen childhood mortalities which is claimed by the authors.  - What kind of strategy was adopted and launched by the government and public in Bangladesh and other developing countries with similar circumstances to improve the rate of receiving PNC? What are the factors they emphasized for influencing mothers to take PNC?  - What are the benefits to build society and nation of the study? Not stated. | Thank you very much for your comments. We revised the Introduction section. | The revised texts are in “red”. Page: 2-4 |
| Methodology:  - Replace “Methodology” with Materials and Methods  - The authors claimed as follows "the choice of variables was influenced by the availability in the BDHS-2017/18 dataset, self-efficacy, as well as relevant literature." What are the literatures?  - What are the criteria for selecting the variables? Did you perform any customization of the variables? If yes, based on what? | Thanks. We change it as per your suggestion. We revised this section. | The revised texts are in “red”. Page: 4-7 |
| Results:  - Move the following sentence in the methods section as it represents method of significance test "Pearson chi-square test was used to identify the covariates whether they were significantly associated with postnatal care or not."  - Sometimes percentage used as %, sometimes as percent. Need to follow uniformity of presentation  - Findings not presented maintaining coherence and cohesion  - What are new in your findings? Are there any new findings than those of in existing literature? Does the result produced by your regression model have similarity or dissimilarity with the findings from logistic regression or any other regression model?  - This section is so redundant as many parts of the tables mentioned in text of this section. | Thank you very much. We moved it to the Methods and Materials section. We also check % and percent and maintain uniformity.  We revised the whole Result section. | The revised texts are in “red”. Page: 7-13 |
| Discussion:  - Start your discussion with providing the answer to your research question  - This section missed some important findings of the study. Need to present only the crucial findings with enough implications of the findings which are lacking  - Literatures that support the study findings are not well articulated for the readers i.e. which settings, what is the actual findings??  - Though study findings showed there some important factors influencing PNC receiving, the authors emphasized on only media campaign, newspaper. Why? Newspaper, how? Is newspaper campaign supported by your findings? | Thanks. We revised the Discussion section. . | The revised texts are in “red”. Page: 13-15 |
| Conclusion:  - This section is also confusing and did not reflect the actual work done  - I think first six sentences of this section are irrelevant here  - The authors stated that they believed that the findings will be beneficial to the policymakers of Bangladesh to lessen the childhood morbidities which will be helpful to achieve the target of the Sustainable Development Goals (SDGs) for lessening preventable maternal as well as under-five deaths by 2030. How? What are your suggestions based on your findings? These are absent. | Thanks for your suggestion. We have revised the manuscript. | The revised texts are in “red”. Page: 15-16 |
| Overall: Language editorial is extensively required. I believe that with appropriate revisions and addressing the concerns raised, this manuscript has the potential to make a valuable contribution to the field. I would recommend inviting the authors to revise and resubmit their work, giving them an opportunity to address the issues raised in this review. | We appreciate this comment. The Methods section has been revised. | The revised texts are in “red”. Page: 1-16 |
| **Reviewer 2 comments:** | **Response** | **Note** |
| The study aims to examine important factors associated with postnatal care in Bangladesh using a secondary dataset extracted from the Bangladesh Demographic and Health Survey 2017-18. Multiple and Multilevel logistic regressions have been used to determine the key factors related to postnatal care. The results are important for policy makers.  The manuscript is systematically written with logical arguments and the evidence are solid to claim the statements. | The authors are thankful to the reviewer for the motivational comments. It motivates young researchers to do research from low-income countries like Bangladesh without any funding support. |  |

In conclusion, the revised version of the manuscript has been produced as per the review outcomes. So, we hope that you will be happy to see this greatly improved version. Once again, we would like to thank you all for your dedication, professional services, and cooperation.
