# Supplementary material for: Determining the influential factors of postnatal care in Bangladesh using multilevel logistic regression
Source: PLoS One. 2024 Nov 7;19(11):e0313424. doi: 10.1371/journal.pone.0313424 (PMC11542818; doi:10.1371/journal.pone.0313424)
Supplement: Attachment — Submitted filename: Review_PONE-D-23-08999.docx [file pone.0313424.s002.docx]

**Summary of the research:**

With an aim to find out influential factors of post-natal care service in the case of Bangladesh, this study used BDHS-2017/18 data, and performed multi-level logistic regression model.

**Overall impression**

The study has undergone 1^st^ stage review and come up in the front with substantial revisions. It is commensurable with other studies using the same data. Its uniqueness is the selection of many explanatory variables and the use of multilevel logistic regression modeling, though the novelty of the research is of question i.e., to what extent the study inform the scientific community.

**Specific comments**

**Major issues**

1. Introduction

The introduction section is still too hazy. Coherence of the literature is missing and the ‘write-up’ is to some extent verbose.

1.1 Several use of, ‘previous study found that’…..is remarkable.
1.2 In the 1^st^ para of the 3^rd^ page, the authors mentioned -“However, most of the studies consider the previous BDHS data and used the logistics regression approach”. There is no indication why they used logistic regression and what are those studies.
1.3 Research question(s) is/are still missing. I suggest the authors to bring research question(s) in the introduction.
1.4 Comparison between global south and global north will be interesting. At least, some comparable literature should be brought from other peer developing countries like Bangladesh.

2. Materials and methods
   2.1 In the specified model in level-1, the authors used error terms. Error terms should be used carefully in in generalized linear model.
   2.2 The model specification is unclear, erroneous. I suggest the authors to check the model specification once again. In the specified model, P_j_ has been referred as an index of independent variables, which actually should be conditional probability. No explanations of η and other symbols used. No source is available from where the model specification has been sought out.
3. Results
   3.1 Performance of the multi-level logistic model has been documented with the use of ROC curve. It is claimed in the manuscript that multi-level logistic model overweighs multiple logistic regression model. But in the legend shows that ‘red colored’ line is multinomial, not multi-level. There is distinction between the two terms. So legend should be checked.
   3.2 The result section is also verbose. I suggest to make it more concise and direct.

**Minor issues**

1. I will suggest the authors to grade the discriminatory performance with the help of guidelines indicated in the book (**Logistic Regression: A Self-Learning Text** written by [David G. Kleinbaum](https://link.springer.com/book/10.1007/978-1-4419-1742-3#author-0-0) , [Mitchel Klein](https://link.springer.com/book/10.1007/978-1-4419-1742-3#author-0-1))
2. Authors are suggested to follow the same format in referencing. In page no. 2, it is written that Mahbub & Zahura (2018)……..while other references have been used in [].
3. In page-5, under statistical methods, it is claimed that “Logistic regression is exploited to determine the impact of socio-demographic and economic factors on postnatal care in Bangladesh”. The word ‘exploited’ should be replaced, and here in this case, with the help of multi-level logistic regression, significantly contributing factors have been identified.

**Overall**

I suggest the authors to be clear and concise in writing the introduction clearly stating research gap and research question(s) once again. The same suggestion is also for the result section. It will be worthy if any help is sought out from anyone who has academic writing skills. I will also suggest to bring change in the manuscript as suggested herewith before publication.
