# Supplementary material for: Determining the influential factors of postnatal care in Bangladesh using multilevel logistic regression
Source: PLoS One. 2024 Nov 7;19(11):e0313424. doi: 10.1371/journal.pone.0313424 (PMC11542818; doi:10.1371/journal.pone.0313424)
Supplement: Attachment — Submitted filename: Review_2_PONE-D-23-08999.docx [file pone.0313424.s003.docx]

**Summary of the research:**

With an aim to find out influential factors of post-natal care service in the case of Bangladesh, this study used BDHS-2017/18 data, and multi-level logistic regression model have been performed.

**Overall impression**

The study has undergone its second-stage review, resulting in several major revisions. While the first part of the introduction still lacks coherence, appearing somewhat disjointed and unclear in its flow, the overall structure and presentation of the study are compelling.

**Issues needs to be considered:**

1. In the introduction, it is claimed that, “Therefore, in order to **prosper** the adverse effects on the child and maternal health, proliferating attention has to be paid to the identification of postnatal care covariates.” The authors are requested to reconsider the use of the word '**prosper**' as it does not align with the intended meaning in this context.
2. In the result section, it us claimed that “True positive rates and false positive rates of fitted models of multilevel logistic regression and logistic regression for different probabilities or marginal values represented by **green** and blue lines, respectively”. Authors are requested to check whether the word **green** should be replaced by the word **red** or not.
3. The sample size varies across several bivariate analyses in Table 1, despite the authors claim in the data section that “After removing missing values, the final sample consisted of 4,729 children under 3 years of age.” It is unclear.

**Overall**

The manuscript is recommended for publication. However, the authors are requested to address the provided recommendations prior to its final submission.
